# Micromechanical Numerical Modelling on Compressive Failure of Recycled Concrete using Discrete Element Method (DEM)

**DOI:** 10.3390/ma13194329

**Published:** 2020-09-29

**Authors:** Xin Tan, Zhengbo Hu, Wengui Li, Suhua Zhou, Tenglong Li

**Affiliations:** 1 College of Civil Engineering, Hunan University, Changsha 410082, China; xintan@hnu.edu.cn (X.T.); huzhengbo@hnu.edu.cn (Z.H.); tenglong_lee@sohu.com (T.L.); 2 Key Laboratory of Building Safety and Energy Efficiency of the Ministry of Education, Hunan University, Changsha 410082, China; 3 School of Civil and Environmental Engineering, University of Technology Sydney, Sydney, NSW 2007, Australia; wengui.li@uts.edu.au

**Keywords:** discrete element method, digital image processing, recycled aggregate concrete, meso-structure, microcrack

## Abstract

This paper investigates the failure processes of recycled aggregate concrete by a model test and numerical simulations. A micromechanical numerical modeling approach to simulate the progressive cracking behavior of the modeled recycled aggregate concrete, considering its actual meso-structures, is established based on the discrete element method (DEM). The determination procedure of contact microparameters is analyzed, and a series of microscopic contact parameters for different components of modeled recycled aggregate concrete (MRAC) is calibrated using nanoindentation test results. The complete stress–strain curves, cracking process, and failure pattern of the numerical model are verified by the experimental results, proving their accuracy and validation. The initiation, growth, interaction, coalescence of microcracks, and subsequent macroscopic failure of the MRAC specimen are captured through DEM numerical simulations and compared with digital image correlation (DIC) results. The typical cracking modes controlled by meso-structures of MRAC are concluded according to numerical observations. A parameter study indicates the dominant influence of the macroscopic mechanical behaviors from the shear strength of the interfacial transition zones (ITZs).

## 1. Introduction

Concrete materials show complex mechanical behaviors because of their internal structures induced by the distribution of different components and weak interfaces at the mesoscopic level [1,2,3]. Recycled aggregate concretes contain various components including natural aggregates, new or old cement mortars, and numerous interfacial transition zones, which extremely increase the complexity of the meso-structure. The meso-structure of recycled aggregate concrete significantly influences the microcrack initiation, propagation and, consequently, macroscopic failure.

A lot of studies have been conducted to investigate the failure behaviors of recycled aggregate concretes using model tests. Buyukozturk et al. [4] firstly proposed an imitated model for plain concrete specimens composed of coarse aggregates and cement mortar to investigate the deformation and failure behaviors. Choi and Shah [5] used modeled concretes to study how the aggregate spacing influences the fracture process of concretes containing different amounts of embedded aggregates. Li et al. [6] used the digital image correlation method to track the cracking process of modeled recycled aggregate concretes (MRAC). Park et al. [7] conducted regression analyses on a substantial amount of data published in Korea and Japan relating to the relationship between the compressive strength and the modulus of elasticity of recycled aggregate concretes. The failure process and crack pattern of the recycled concrete specimens were strongly affected by the stiffness difference between the recycled coarse aggregate and cement mortar. Ren et al. (2015) [8] built a 2D finite element model of concretes with realistic meso-scale structures using microscale X-ray CT images. Shang et al. (2020) [9] used micro-CT images to analyze the failure cracks of recycled aggregate concrete in the laboratory. Their experimental and numerical results show that the meso-failure mode of coarse aggregate concrete is different from that of normal concrete, which depends on the interfacial transition zone (ITZ) between the coarse aggregate and the cement paste and the properties of the recycled coarse aggregate (RCA) itself.

Recently, numerical simulations verified by experimental results have become a powerful research methodology to better understand the complex failure mechanism of concrete. Several numerical studies adopted plasticity–damage constitutive models have shown good correlations with the experimental results [10,11]. Some researchers simulated the meso-structure of concretes by continuum finite elements, which discretize the aggregates and cement with the interfaces characterized by weaker zones or interface elements [12,13]. Yang et al. [14] simulated mesoscopic cracking in concretes using X-ray computed tomography image-based modeling. Yang et al. [15] presented a comprehensive review study of the numerical methods developed for modeling heterogeneous materials such as cement concretes and asphalt mixtures.

Concretes are usually idealized as a continuous material defined by certain constitutive relations [16,17] by continuum-based numerical methods, which have some limitations to capture the microcracking process. The dis-continuum based methods have an advantage over the continuum methods in simulating the microcracking process of heterogeneous materials by introducing the contact models to directly represent the mechanical behaviors between the cemented granular particles [18]. A lot of numerical simulation works based on the Discrete Element Method (DEM) have been conducted successfully to simulate the microscopic damage process of rocks [19,20,21,22]. Concretes and rocks have very similar mechanical characteristics; therefore, the DEM model could be an ideal tool to simulate concretes. The numerical simulation from Schubert et al. [23] shows the cracking phenomena of concretes composed by various-sized aggregates can be well captured by the DEM model. Tan et al. [24] conducted DEM simulation on the failure process of recycled aggregate concrete under uniaxial compression, whose numerical results show very similar results to the model test observations.

Micromechanical modeling based on the DEM method has been proposed in this study to simulate the progressive cracking behavior of the modeled recycled aggregate concrete, considering its actual meso-structures. By correlating the experimental results, the influence of different components on the cracking process of the modeled recycled aggregate concrete can be investigated. The complex cracking behaviors of the modeled recycled aggregate concrete specimen during the complete failure process is governed by the initiation, growth, interaction, and coalescence of microcracks.

## 2. Experimental Program

### 2.1. Specimen Preparation

**Figure 1:**
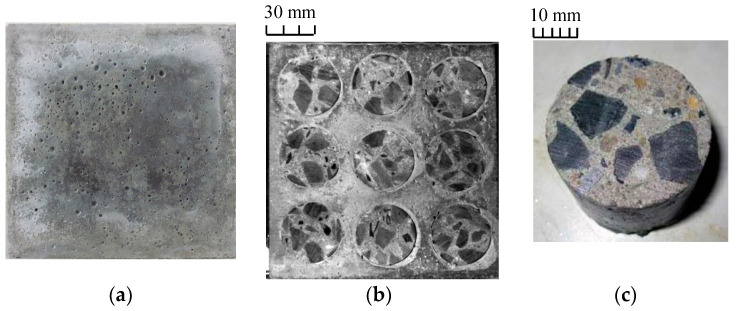
(**a**) cement mortar; (**b**) modeled recycled aggregate concrete; (**c**) recycled aggregate.

The modeled recycled aggregate concrete specimen (MRAC) is prepared to represent the recycled aggregate concrete in the laboratory. As shown in Figure 1, the MRAC specimen contains 9 recycled aggregate models, which are idealized as cylindrical inclusions. The recycled aggregate inclusions of diameter 38 mm were cored from the old concrete. The dimensions of the cement mortar matrix, which models the new cement mortar in the recycled aggregate concrete, are 150 mm × 150 mm × 30 mm. The MRAC specimen had been immersed in limestone-saturated water for 28 days for curing. A specimen of the pure cement mortar (CM) with the same dimensions has been prepared for comparison. The particular specimen preparation method has been documented by previous studies [8,25].

### 2.2. Nanoindentation Test

**Figure 2:**
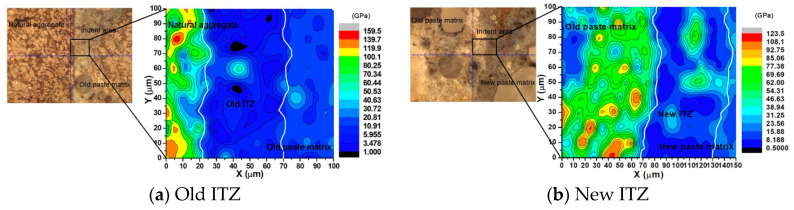
Nanomechanical properties of interfacial transition zones (ITZs). (**a**) Old ITZ (**b**) New ITZ.

**Figure 3:**
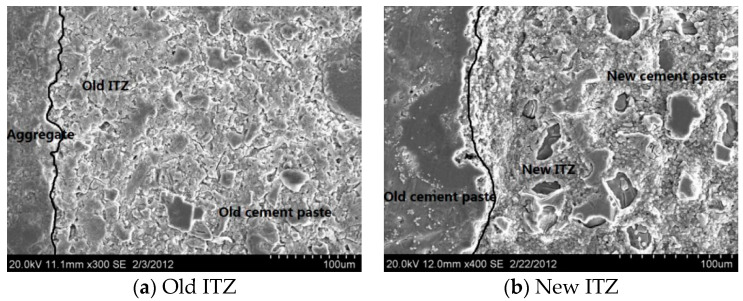
SEM images of ITZs. (**a**) Old ITZ (**b**) New ITZ.

It is well recognized that the cracking and failure process of concretes is strongly influenced by the interfacial transition zones (ITZ). Therefore, the mechanical and microstructural properties of ITZs in RAC were investigated by using nanoindentation tests and scanning electron microscopy (SEM). Two indentation areas with a size of 150 μm × 100 μm were located within the old ITZ and new ITZ region, as shown in Figure 2. The contour maps of the indentation modulus of old and new ITZs are obtained from nanoindentation tests. The darker areas mean a lower modulus region, while the brighter areas mean higher modulus regions. Figure 2 clearly shows the modulus difference in different portions, which indicates that the microstructure in the ITZ region is quite different from that of the mortar matrix. Figure 2a shows that the indentation modulus of old ITZs is around 70%~80% of the old mortar matrix, and Figure 2b shows that the indentation modulus of new ITZs is around 80%~90% of the new mortar matrix. The thickness of ITZs is around 40~70 μm. There are many more voids that can be observed in the ITZ region from the SEM images (Figure 3), which leads to the lower stiffness and strength of the ITZs. The indentation modulus distributions of different components of the MRAC specimen provide the reference for the quantitative analysis of the microscopic parameters in numerical modeling.

### 2.3. Uniaxial Compression Test and DIC Measurement

**Figure 4:**
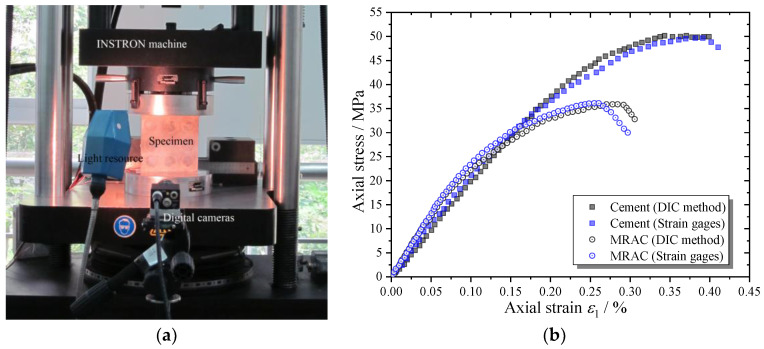
Setup for compression test and digital image correlation (DIC) measurements (**a**) equipment; (**b**) complete compressive stress–strain curves of cement mortar (CM) and MRAC.

The MRAC and CM specimens were uniaxially compressed under a minimal loading rate by the serve-control machine. During the complete loading process, the cracking process of the specimen was captured by digital image correlation (DIC) measurement [8,25] (Figure 4). The microcracks can be recognized according to the image analysis on the displacement or strain distribution images taken from the specimen. The axial strain of the specimen was measured by both the DIC method and conventional strain gages, which are compared and shown in the complete stress–strain curves in Figure 4b. The other DIC measurement results including displacement and strain distribution contours and failure patterns are compared with the numerical results in the following section.

## 3. Numerical Simulation

### 3.1. DEM and UDEC Program

Cundal and Strack [18] introduced the original idea of Discrete Element Method (DEM) to calculate the motion of a block system by the explicit time-marching scheme. Since then, DEM approaches have been widely adopted to simulate fracture processes in mechanical rock problems [26]. In contrast to continuum-based numerical models, which need hypothetical constitutive relations to simulate material behaviors, DEM models use discrete particles or blocks represent actual physical objects like grains in granular rocks or aggregates in concretes. All the discontinuities inside the materials, such as fractures, cracks, and pores, are inherent parts of the DEM model. Therefore, DEM models are particularly favorable for investigating the cracking processes of brittle heterogeneous materials.

**Figure 5:**
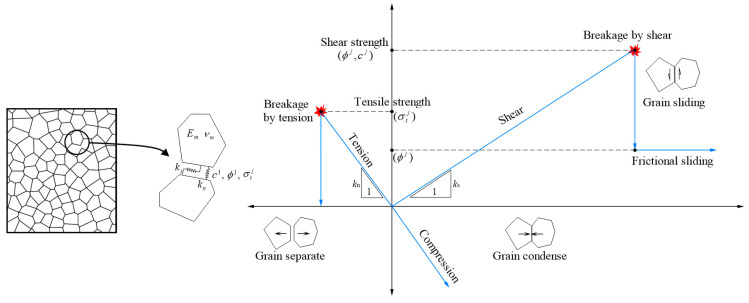
Voronoi model with the indication of contact micro-parameters and force–displacement and failure behavior for the contact between two blocks [24].

The universal distinct element code (UDEC) program is a 2D numerical code under the framework of the DEM concept. UDEC can simulate either static or dynamic responses of a discontinuous system composed of either rigid or deformable discrete blocks. As shown in Figure 5, a plane concrete specimen can be modeled as the assembly of discrete blocks with random sizes and shapes in UDEC. The Voronoi tessellation procedure is introduced to subdivide the concrete specimen into randomly sized polygons. The Voronoi blocks can be either rigid or subdivided into elastic elements with Young’s modulus $E^{g}$ and Poisson’s ratio $\nu^{g}$. The contacts between the blocks are deformable and breakable, which is represented by the Coulomb slip model with tensile strength, as shown in Figure 5. The following contact parameters are involved to calculate the force–displacement relationship of a contact: normal stiffness ($k_{n}$), shear stiffness ($k_{s}$), friction angle ($\phi^{c}$), cohesion ($c^{c}$), and tensile strength ($\sigma_{t}^{c}$). A microcrack is generated when the contact breakage happens by shear or tension. Therefore, the block boundaries provide numerous potential internal paths for the growth of microcracks. Macroscopic fractures can eventually be formed due to the initiation, propagation, and connection of microcracks. Tan et al. [24] presented a numerical analysis of the failure process of modeled recycled aggregate concrete using the UDEC program, whose results showed very similar observations as the experimental results.

### 3.2. Image Based Meso-Structures Model and Microscopic Contact Parameter Determination

**Figure 6:**
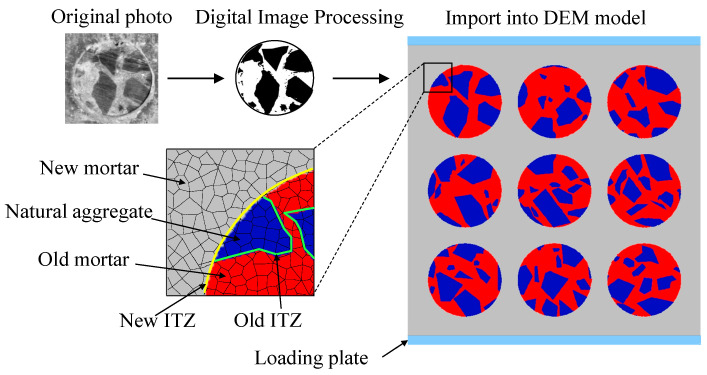
Image based meso-structure discrete element method (DEM) model of modeled recycled aggregate concrete (MRAC) specimen.

The UDEC program was adopted to simulate the MRAC specimen and analyze the cracking evolvement and progressive failure process of the specimen, considering meso-structures. As described in the last section, the MRAC specimen was simulated by discrete Voronoi blocks. The actual MRAC specimen consists of several different components at the meso-level scale that may influence its overall mechanical behaviors, including natural aggregates, new and old cement mortar, and new and old ITZs. In particular, the ITZs with the lowest physical strength may be the critical factor in controlling the microcracking process of the MRAC specimen. To model the meso-structures of the MRAC specimen, Digital Image Processing (DIP) is introduced, as illustrated in Figure 6. Binarization processing was used to transform the digital image of the MRAC specimen into a black and white image. The contrast between the old mortar matrix and the natural aggregate can be easily observed from the black and white image. The boundaries of each natural aggregate can then be extracted as polylines from the image by the outline identification technique. The concrete specimen was subdivided into abundant convex-shaped blocks of random size by the Voronoi tessellation procedure. The Voronoi blocks in the numerical specimen were then divided into three groups according to the boundaries recognized from the digital image, which are the new mortar matrix, the old mortar matrix, and the natural aggregate. The boundaries between different groups are therefore the ITZs.

The ITZs were modeled in the DEM model as zero-thickness contacts and endowed with corresponding contact parameters according to the coordinates of the extracted polylines, while, in the continuum numerical models (Anuruddha et al. 2018 [13]), the thickness of ITZs was considered from 0.5~2.0 mm, which is obviously much higher than the thickness of 40~60 μm measured from nanoindentation tests [27,28]. The old mortar and the aggregate portions can also be distinguished depending on which one is internal and which is external of the ITZ polylines. Rigid blocks are assumed in the proposed model. Thus, the mechanical behaviors of the MRAC specimen are completely controlled by its microscopic contact parameters. Three different groups of contact parameters are needed to model the MRAC specimen, which are natural aggregates, cement mortar, and ITZs, respectively. To simplify the parameter calibration procedure, the mortar and ITZs were not distinguished into old or new groups.

The contact parameters for different components in the MRAC specimen must be determined firstly on the condition that the macroscopic mechanical behaviors of the simulated material such as deformability, uniaxial compression strength, and failure pattern can be generally reproduced. Nevertheless, the microscopic contact parameters are diverse and different from the regular parameters obtained from experiments [21,22]. A determination procedure has to be performed in advance to determine proper contact parameters according to the macroscopic mechanical behaviors of the DEM model.

There is only one group of contact parameters needed for the CM specimen model; therefore, the contact parameters of mortar can be determined firstly by comparison with the uniaxial compression results of the CM specimen. Tan et al. [29] have provided empirical relations to calculate the contact stiffnesses $k_{n}$ and $k_{s}$ based on the elastic modulus and Poisson’s ratio of the Voronoi-based DEM model. The contact parameters ($\phi^{c}$, $c^{c}$ and $\sigma_{t}^{c}$) of the CM specimen can be predefined according to their corresponding macroscopic strength parameters. The peak strength and corresponding axial strain in the compressive stress–strain curve of the CM specimen (Figure 8) are the target parameters to calibrate the contact parameters.

**Figure 7:**
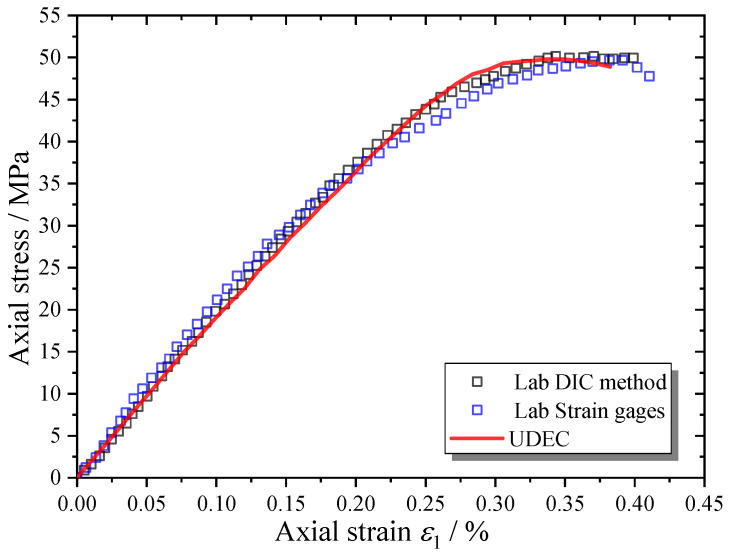
Complete compressive stress–strain curves of CM specimens.

**Figure 8:**
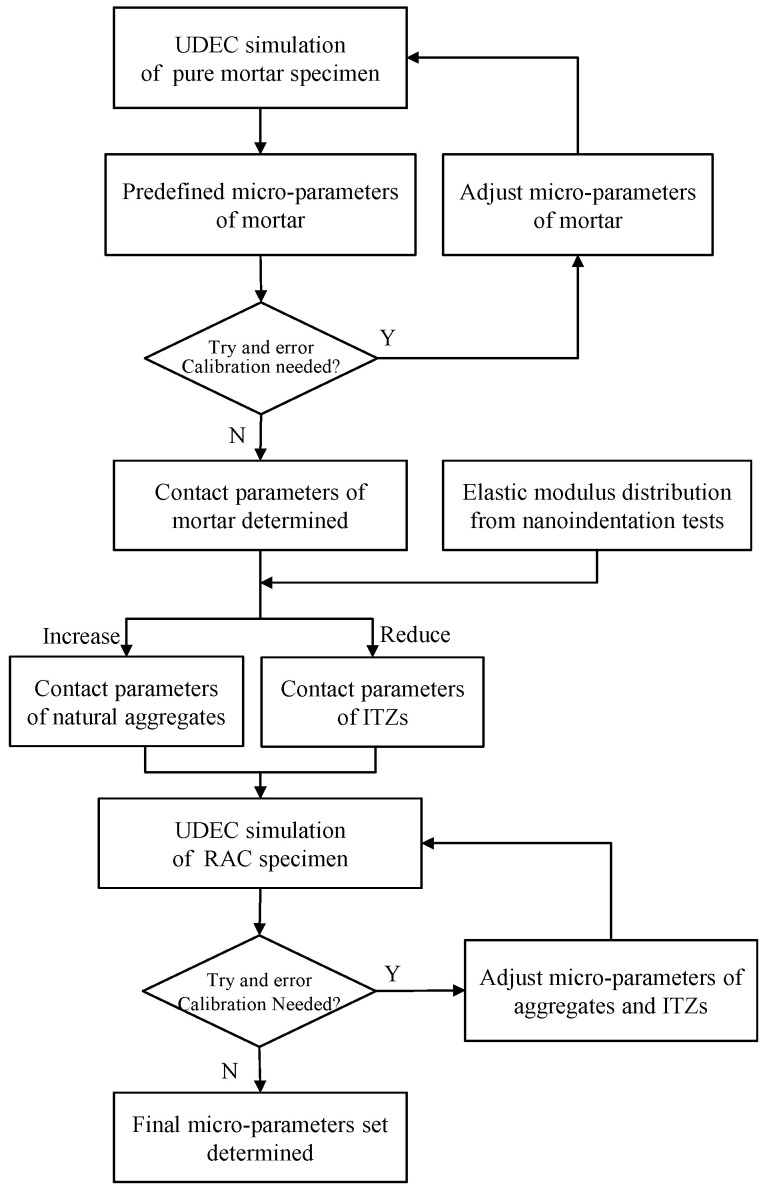
Micro-parameters determination procedure for MRAC simulation.

**Figure 9:**
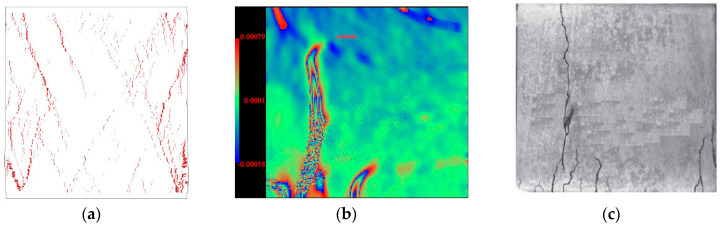
Microcracks distribution in CM at failure state (**a**) numerical results; (**b**) DIC measurement; (**c**) fracture pattern.

Then, the contact parameters can be further modified according to several trials and errors. The determination flow diagram is illustrated in Figure 7. The complete stress–strain curves and failure patterns of the numerical CM specimen with calibrated contact parameters are shown in Figure 8 and Figure 9 in comparison with the experimental results.

The numerical result in Figure 9a shows more cracks and main fractures compared to the experimental results in Figure 9b,c. The main reason for these discrepancies is that the numerical CM specimens are almost homogeneous, which leads to a multi-fracture pattern at the failure state. In reality, the location where the main fracture appears depends on the material flaws inside the CM specimen. Moreover, the numerical model (Figure 9a) is able to show much more details compared to the experimental observations, including micro-cracks with an extremely small crack width. However, the real photo in Figure 9c can only show the main fractures with relatively wider apertures. The DIC picture in (Figure 9b) can show the micro-cracks to a certain degree, but not precisely. Nevertheless, the typical cracking behaviors are actually captured by the numerical model.

The contact parameters of other components can then be predetermined by taking the contact parameters of the CM specimen as a standard. The microhardness obtained from the nanoindentation test provides a quantitative scale between the contact stiffness and strength for different components in concrete [30,31]. Still, trials and errors are always necessary before optimum contact parameters are obtained. An entire set of contact parameters for the MRAC specimen has been determined and is listed in Table 1. The loading plate was modeled using the linear elastic constitutive law.

A sufficient number of discrete blocks should be contained in a DEM model to avoid significant uncertainty in the macroscopic mechanical behaviors. The average block edge length is set to 1.0 mm, as the DEM specimen contains 12,859 blocks with an average block area 1.8 × 10^−6^ m^2^ and 62,500 contacts. The axial stress acting on the specimen was controlled by moving the two loading plates in opposite directions. The loading velocity was kept at a small value so that the cracking process could be well recorded during the complete loading process.

## 4. Numerical Analysis

### 4.1. Stress–Strain Curve and Contact State Evolvement

**Figure 10:**
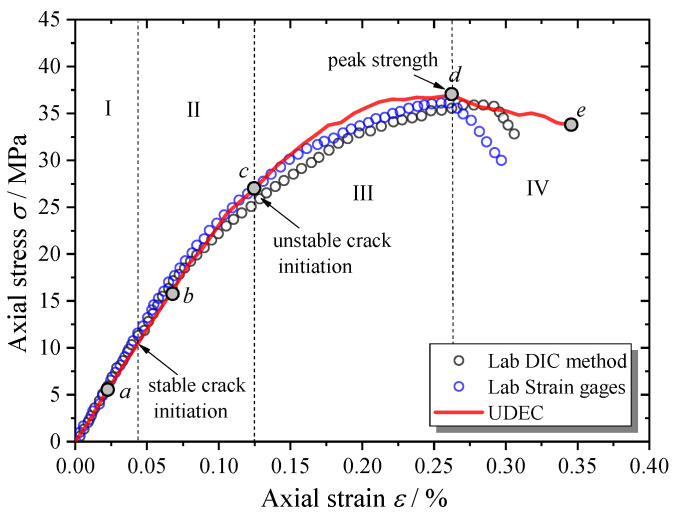
Complete compressive stress–strain curves of MRAC specimen.

**Figure 11:**
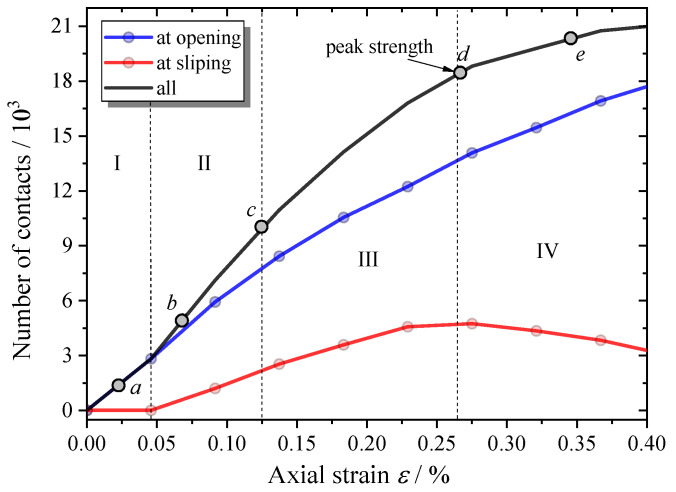
Microcrack evolution during the complete failure process.

The complete stress–strain curve of the MRAC specimen predicted by the numerical simulation is shown in Figure 10, which is in good agreement with the laboratory data. In particular, in the pre-peak loading stages, a very similar curve shape and almost the same macroscopic strength can be observed. The numbers of contacts in tensile opening and shear sliding during the complete loading process of the simulation are shown in Figure 11, which represents the microcrack evolution inside the MRAC specimen. Four typical stages in the cracking process are labeled with Roman numerals in Figure 10 according to the stress–strain curve and microcrack evolution. Stage (I): linear elastic deformation stage; Stage (II): stable cracking stage; Stage (III): unstable cracking stage; and Stage (IV); post-failure stage.

### 4.2. The Complete Failure Processes

**Figure 12:**
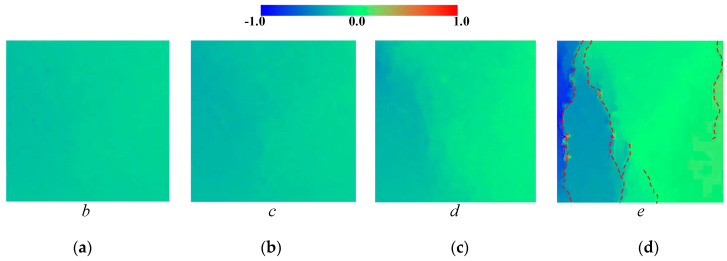
Lateral displacement distribution contour maps from DIC measurements (unit: mm) (**a**) loading stage *b*; (**b**) loading stage *c*; (**c**) loading stage *d*; (**d**) loading stage *e*.

**Figure 13:**
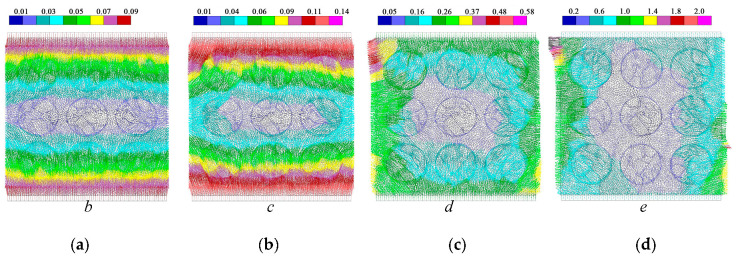
Displacement vectors of block corners from numerical simulation (unit: mm) (**a**) loading stage *b*; (**b**) loading stage *c*; (**c**) loading stage *d*; (**d**) loading stage *e*.

The deformation field and crack distribution of the MRAC specimen from both DIC measurements and numerical simulations at the selected loading stages (marked in Figure 10) are compared to each other. Figure 12 and Figure 13 show the macroscopic deformation field of the MRAC specimen at different loading stages (b∼e). Figure 12 shows the lateral displacement distribution of the MRAC specimen obtained by DIC measurements. Several macroscopic fractures were observed for MRAC specimens after failure according to the discontinuity in the lateral displacement contour. The fracture directions are almost vertical. Figure 13 shows the displacement vectors of the block corners of the MRAC specimen obtained by numerical simulations. Similar macroscopic fractures of the MRAC specimen at stage e are marked in Figure 13 according to the discontinuity in the displacement.

**Figure 14:**
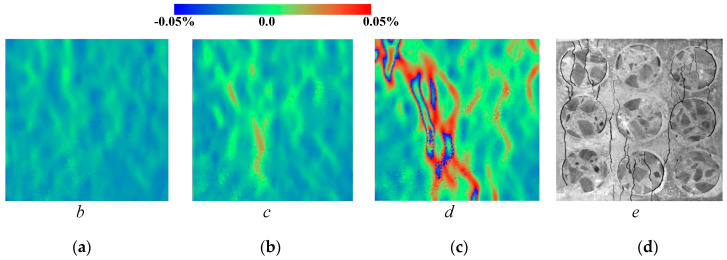
Lateral strain distribution contour maps from DIC measurements (**a**) loading stage *b*; (**b**) loading stage *c*; (**c**) loading stage *d*; (**d**) loading stage *e*.

**Figure 15:**
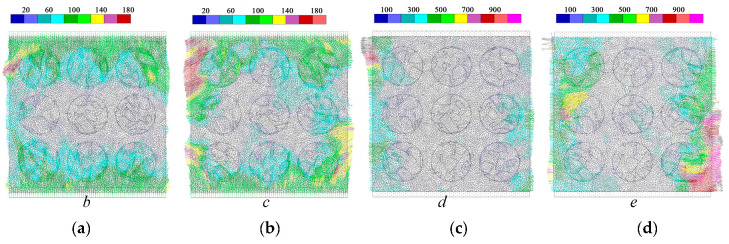
Velocity vectors of block centroid from numerical simulation (unit: mm/sec) (**a**) loading stage *b*; (**b**) loading stage *c*; (**c**) loading stage *d*; (**d**) loading stage *e*.

Figure 14 shows the lateral strain distribution of the MRAC specimen obtained by DIC measurements and a photo of the final fractured pattern. The strain distribution contour shows much more evident localization of deformations than the displacement contour, which reflects the microcracking evolvement of the MRAC specimen during the complete loading process. It is observed that microcracks appear mostly in the ITZs and the mortar region at stage b. Vertical cracks mainly originated from the ITZs, as the loading increases at stage c the cracks propagated in the mortar region. A large number of observable microcracks were generated and formed a connected crack–band at peak loading stage d. The crack–band is slightly different from the final fractured pattern at the post-peak loading stage e, which means some macro-fractures are generated after the peak strength of the specimen. The deformation localization can also be observed in the numerical results according to the velocity vectors of block centroid in Figure 15. Very similar fracture patterns are found in both experimental and numerical results at stage e, which indicates the mesoscopic heterogeneities of the MRAC specimen is well represented by the proposed numerical model.

**Figure 16:**
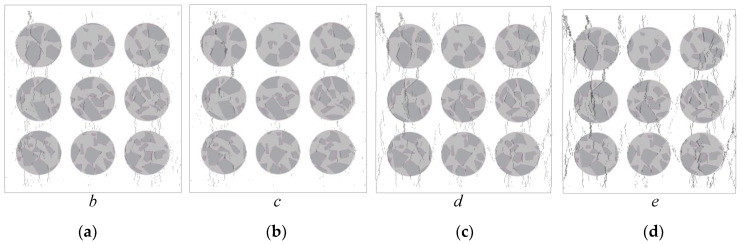
Tensile crack distributions at different loading stages from numerical simulation (**a**) loading stage *b*; (**b**) loading stage *c*; (**c**) loading stage *d*; (**d**) loading stage *e*.

**Figure 17:**
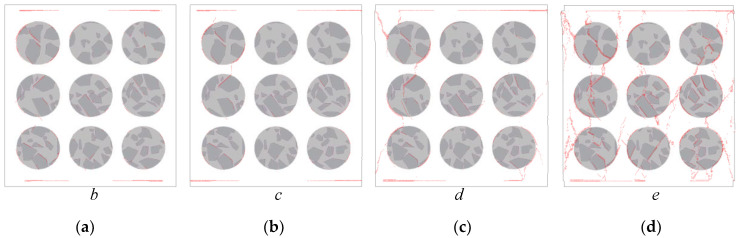
Shear crack distributions at different loading stages from numerical simulation (**a**) loading stage *b*; (**b**) loading stage *c*; (**c**) loading stage *d*; (**d**) loading stage *e*.

The DIC measurement captured the lateral displacement and lateral strain on the MRAC specimen surface, which reflects the cracking propagation of the MRAC specimen during the loading process. The DEM model can provide further information about the cracking mode of each microcrack since either the tensile and shear contact failure mechanism is in consideration as shown in Figure 5. The contact breakages caused by the tensile failure and the shear failure are plotted in Figure 16 and Figure 17. The width of contacts indicates the separation and sliding displacement of contacts, respectively. There are some horizontal shear cracks in Figure 17, which represents the shear displacement between the specimen and the loading plates due to the Poisson effect.

**Figure 18:**
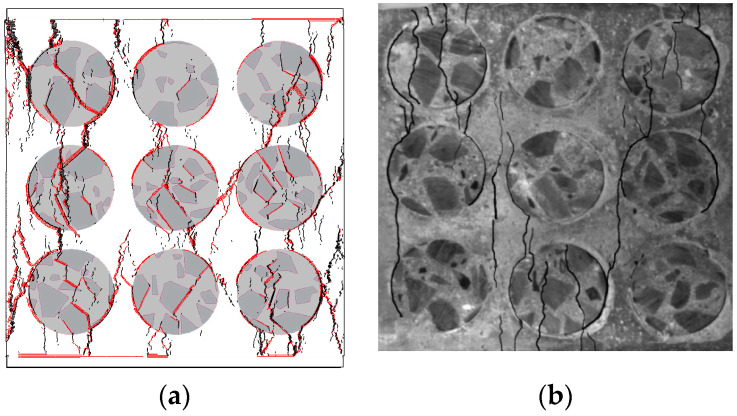
Crack distribution at stage *e* (post-peak loading stage): (**a**) numerical results; (**b**) fracture pattern.

A great number of tensile microcracks are found at stage b, which is far away from the peak loading stage. A lot of tensile cracks randomly initiate in the mortar region in the loading direction (stage b in Figure 18). Moreover, some tensile cracks are generated along the ITZs, which have small angles (<10°) relative to the loading direction. The shear cracks seem to be more dependent on the meso-structures compared to the tensile ones. Almost every shear crack initiate along the ITZs (stage b in Figure 17), which have a certain angle (30° < 60°) relative to the vertical direction.

As the loading increases, more and more tensile cracks grow and propagate in the loading direction. A few tensile cracks concentrate along the vertical ITZs, and in the mortar regions where between an upper and a lower aggregate (stage c in Figure 16). Some tensile cracks and shear cracks begin to be connected as they are continuously forming and propagating at stage *c*. Some macroscopic fractures, therefore, appear and lead to the localization of deformation observed in Figure 14. These fractures do not penetrate the whole MRAC specimen; therefore, the stress–strain curve in Figure 10 does not lose the load-carrying capability but shows a nonlinear shape at stage c.

The fracture keeps growing due to the connecting of microcracks during the loading process. Finally, a predominant macroscopic fracture penetrates through the whole MRAC specimen at stage d, and peak loading is then achieved. The loading may suddenly distribute on different fragments of the ruptured specimen, which creates more macroscopic fractures after the peak load, as shown in stage *e* in Figure 16 and Figure 17.

Moreover, the overlays of Figure 16 and Figure 17 of stage *e* are compared with the failure specimen, as shown in Figure 18. Due to the limitation of the 2D model, the real 3D meso-structure of the concrete specimen cannot be fully modeled in the proposed model. The spatial distribution of aggregates inside the concrete specimen was not captured. Therefore, the numerical model is not able to capture all the cracks or fractures observed from experimental results in every detail. Nevertheless, the numerical results show quite good correlation with experimental results, based on both the stress–strain curves and the crack distributions.

### 4.3. Cracking Modes

**Figure 19:**
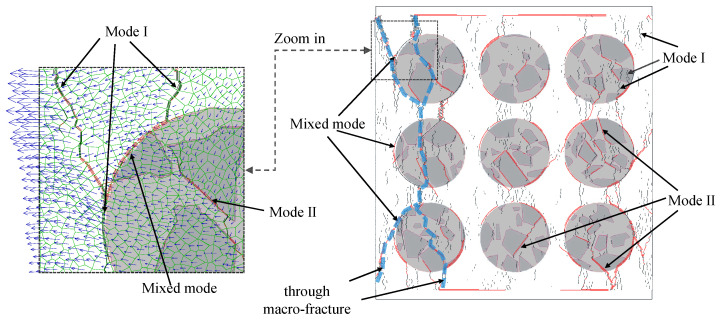
Enlarged view of crack distribution at stage *d* (peak loading stage).

According to the analysis in the last section, the initiation and evolution of microcracks are dependent on the cracking modes, which will control the final failure behaviors of the MRAC specimen. The mode I (tensile microcracks) and mode II (shear microcracks) cracks at the peak loading stage are thus plotted in a single figure (Figure 19). The influence on the cracking modes from the mesoscopic structure of the MRAC specimen can be observed. Either mode I or mode II cracks can be found in the new mortar region. However, most of them are mode I cracks. A small amount of mode II cracks was generated after the macroscopic fractures formed. The cracks of mode I widely distribute in the new mortar region and angle less than 10° from the loading direction. The cracks of mode I, with large openings, are found to appear mainly between an upper and a lower natural aggregate, where the compressional stress concertation always happens. The cracks of mode I can also be observed in the recycled aggregate region, where they are mostly inside the old mortar region and in the old ITZs, with directions nearly parallel to the loading direction. Because of the high strength of the natural aggregates, none of the mode II cracks are observed in the natural aggregate region. Very few cracks of mode I are found, apart from some particular ones with long, narrow shapes and a long axis perpendicular to the loading direction.

The mode II cracks appear mostly along the old ITZs inside the recycled aggregate region, which has angles larger than 10° in the loading direction. While outside the recycled aggregate region, both mode I and mode II cracks can be observed along some new ITZs. These cracks, therefore, can be defined as mixed mode cracks.

**Figure 20:**
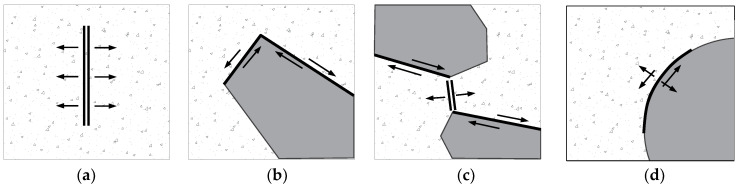
Schematic diagram of different cracking modes: (**a**) compression induced tensile crack; (**b**) shear crack; (**c**) shear induced tensile crack; (**d**) mixed crack.

The typical cracking modes controlled by their corresponding mesoscopic structures revealed in the numerical simulation are schematically illustrated in Figure 20. Although the cracks of mode I are dominant in quantity, as shown in Figure 11, the final macroscopic fractures are generated due to the propagation and the connection of all different modes of cracks. The failure process of the simulated MRAC specimen can be considered as a self-organizing process of the microcracks. The process is dependent on the spatial distribution of different components of the MRAC specimen at the mesoscopic scale.

## 5. Parameter Study and Discuss

The macroscopic failure process of the MRAC specimen is closely related to microcrack development during the complete loading process. Therefore, variations in the mechanical properties of microscopic contacts ultimately affect the macroscopic mechanical behaviors. Some key microscopic contact parameters are selected for further parameter analysis.

**Figure 21:**
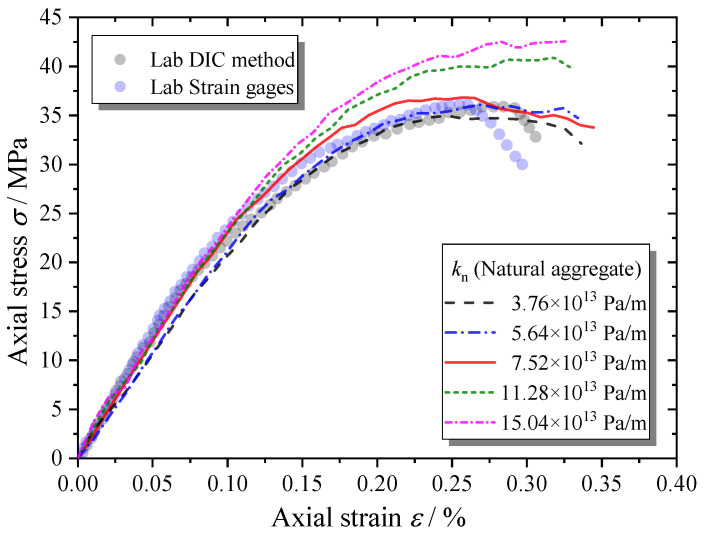
Complete compressive stress–strain curves of MRAC specimen with various natural aggregate stiffness values.

The microcracks can hardly be observed inside the natural aggregates during the loading process. It is believed that the natural aggregates have enough tensile and shear strength, which will not strongly influence the macroscopic behaviors of the MRAC specimen. Thus, only the normal stiffness of contact $k_{n}$ of natural aggregates has been tested for parameter analysis. As shown in Figure 21, the macroscopic stiffness of the MRAC specimen increases with the increase in natural aggregate stiffness, but not evidently. The loading level to start the unstable cracking stage became higher for stiffer natural aggregate cases, which led to higher peak strength.

**Figure 22:**
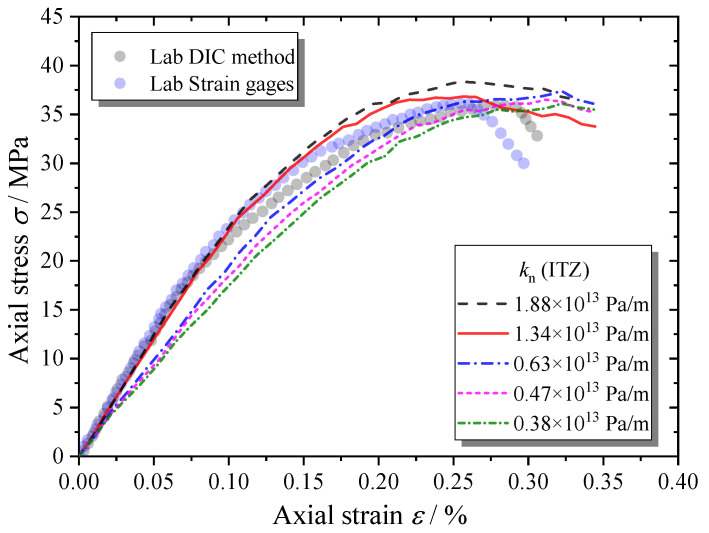
Complete compressive stress–strain curves of MRAC specimen with various ITZ stiffness values.

Figure 22 shows that the contact stiffness of ITZs has a greater influence on the macroscopic stiffness of the MRAC specimen than the stiffness of the natural aggregate, while it has little effect on the peak strength.

**Figure 23:**
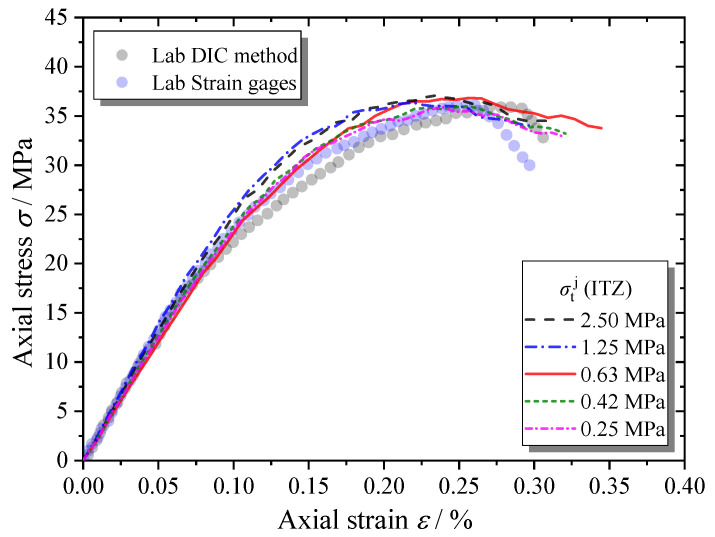
Complete compressive stress–strain curves of MRAC specimen with various ITZ tensile strength values.

Figure 23 shows that the tensile strength of ITZs seems to have little influence on the macroscopic mechanical behaviors of the MRAC specimen. As we discussed in the last section, the cracks of mode I only appear in the ITZs that are nearly parallel to the loading direction. Tensile failure does not control the cracking process of the ITZs.

**Figure 24:**
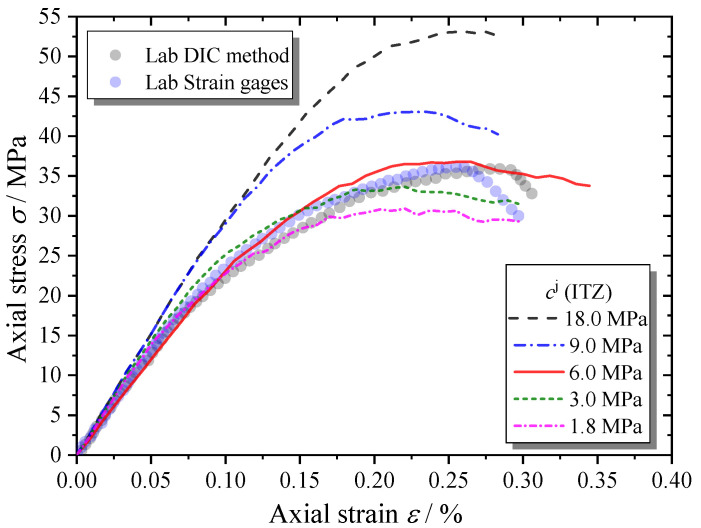
Complete compressive stress–strain curves of MRAC specimens with various ITZ shear strength values.

Unlike the tensile strength, the shear strength of the ITZs shows a significant influence on the macroscopic mechanical behaviors of the MRAC specimen, as shown in Figure 24. Because the failure modes of most ITZs are mode II or mixed mode, as shown in Figure 19, the increase in the shear strength of the ITZs can significantly increase the peak strength and the loading levels to start both the stable and the unstable cracking stages. The shear strength of the ITZs needs to be very carefully considered in DEM numerical models.

## 6. Conclusions

The failure process of the MRAC specimen under a uniaxial compression test was investigated using both experimental and numerical methods. The actual heterogeneity of the modeled concrete specimen contains different components and ITZs can be well reproduced by combining the DEM model and DIP method. The main conclusions drawn from the proposed study are summarized as follows:

(1) The simulated cracking and failure process of the MRAC specimen shows very similar observations to the experimental results. The proposed numerical model provides the possibility to investigate the failure mechanism of the recycled aggregate concrete at a microscopic level, which is beyond the ability of continuum approaches.
(2) The failure process of the MRAC specimen can be considered as a self-organizing process of microcracks and is dependent on the mesoscopic structure of the specimen. The typical cracking modes controlled by the corresponding mesoscopic structures are concluded according to the numerical observations.
(3) The parameter study shows that the shear strength of the ITZs has a dominant influence on the macroscopic strength of the recycled aggregate concrete, which needs careful consideration in the DEM models.

## Figures and Tables

**Table 1:** Micro-mechanical parameters used in numerical simulations.

Material Type	*k*_n_	*ks*	*ϕ* ^j^	*c* ^j^	*σ*_t_^j^	*E*_m_	*ν*_m_
		Pa/m	°	MPa	MPa	GPa	/
Natural aggregate	7.52 × 10^13^	3.76 × 10^13^	35	50.0	10.0	/	
Mortar ITZ	1.68 × 10^13^	9.40 × 10^12^	10	18.0	2.5		
	1.34 × 10^13^	3.35 × 10^12^	24	6.0	0.63		
Loading plate						200	0.15
